# Awareness and Use of Low-Sodium Salt Substitutes and Its Impact on 24-h Urinary Sodium and Potassium Excretion in China—A Cross-Sectional Study

**DOI:** 10.3390/nu15133000

**Published:** 2023-06-30

**Authors:** Puhong Zhang, Fang Fan, Yinghua Li, Yuan Li, Rong Luo, Li Li, Gang Zhang, Lanlan Wang, Xiaofei Jiao, Feng J. He

**Affiliations:** 1 The George Institute for Global Health, Beijing 100600, China; zpuhong@georgeinstitute.org.cn (P.Z.);; 2 The George Institute for Global Health, Faculty of Medicine, University of New South Wales, Sydney, NSW 2052, Australia; 3 School of Public Health, Anhui Medical University, Hefei 230032, China; 4 Chinese Center for Health Education, Beijing 100011, China; 5 Wolfson Institute of Population Health, Barts and The London School of Medicine & Dentistry, Queen Mary University of London, London E1 4NS, UK; f.he@qmul.ac.uk

**Keywords:** low-sodium salt substitutes (LSSS), 24-h urinary sodium excretion, 24-h urinary potassium excretion, sodium-to-potassium ratio

## Abstract

The use of low-sodium salt substitute (LSSS) has the potential to reduce sodium and increase potassium intake. LSSS has been available in the Chinese market for years. However, its real-world use and impact on sodium/potassium intake is unclear. Baseline data of 4000 adult individuals who participated in three similarly designed randomized controlled trials were pooled together for this analysis. Self-reported awareness and use of LSSS were collected using a standardized questionnaire, and the participants’ 24-h urinary sodium and potassium excretion was used to estimate their dietary intake. Mixed-effects models were developed to assess the relationship between LSSS and 24-h urinary sodium and potassium excretion. 32.0% of the participants reported awareness of LSSS and 11.7% reported its current use. After adjusting for location, sex, age, and education, compared with the group of participants unaware of LSSS, participants who were aware of but not using LSSS and those who were using LSSS had a lower 24-h urinary sodium excretion by −356.1 (95% CI: −503.9, −205.9) mg/d and −490.6 (95% CI: −679.2, −293.7) mg/d, respectively (*p* < 0.001). No significant difference was found for 24-h urinary potassium excretion or sodium-to-potassium ratio among the three groups (*p* > 0.05). In conclusion, the findings of low usage of LSSS and the reduced urinary sodium excretion associated with the awareness and use of LSSS provide further support for the prometon of LSSS as a key salt reduction strategy in China.

## 1. Introduction

In China, more than 2.4 million people die of cardiovascular disease every year, which is the main cause of death among Chinese residents [1], and hypertension is one of the main risk factors for cardiovascular disease. Evidence has shown that excessive salt intake and insufficient potassium intake were both crucial dietary factors in raising blood pressure [2,3,4]. According to the survey, more than 1.5 million deaths each year can be attributed to high-salt diet, which has become the third risk factor for death and the first risk factor in diet [1]. WHO recommends that adults consume <2000 mg/d sodium and >3510 mg/d potassium to reduce the risk of chronic diseases [5,6]. However, due to traditional dietary habits, individuals consume more than twice of the recommended sodium intake and less than half of the recommended potassium intake in China [7].

Several studies have supported the claim that the use of low-sodium salt substitutes (LSSS) was an effective intervention to reduce dietary salt intake and increase potassium intake [4,8]. In a trial in rural areas of northern China, after five years of follow-up, it was found that the mean 24-h urinary sodium excretion was reduced by 350 mg and the 24-h urinary potassium excretion was increased by 803 mg in the LSSS group compared to the normal salt group [9]. A review showed that LSSS compared to regular salt probably increases blood potassium slightly 0.12 mmol/L, and reduces diastolic blood pressure and systolic blood pressure by an average of 2.43 mmHg and 4.76 mmHg, respectively [10]. LSSS reduces the amount of sodium chloride, most commonly by replacing it with potassium chloride or magnesium sulfate [11]. The composition of LSSS may vary from country to country due to taste and acceptability [12,13]. For example, LSSS produced in India contains less than 20% potassium chloride, while in countries in North America, the Middle East, and Latin America, potassium chloride accounts for at least 50%, and in the United States and Canada, it may account for 100% (sodium-free) [14,15]. The proportion of potassium chloride in LSSS is from 25% to 30% in China [15].

A series of policies to reduce salt intake have been introduced by the Chinese government [16]. At the national level, the third week of September is set as National Salt Reduction Week, focusing on publicizing low-salt diet knowledge to residents and advising all residents to pay attention to and implement salt reduction actions. In addition, salt reduction has been taken as a core theme in the annual National Nutrition Week [17] and China Healthy Life for All campaign [18], and a series of health education activities have been carried out around it. In the Healthy China Action [16], it is clearly proposed to encourage enterprises to produce and sell LSSS, and to promote the use of LSSS under the guidance of experts. The Action also proposes pilot work to promote LSSS in areas where conditions permit [16]. Regionally, some measures have been taken to promote LSSS in Beijing in 2009, such as setting up designated areas in supermarkets or shops for foods with less salt, and rewarding consumers with an additional 75 g of LSSS for every 400 g they purchased [19]. Moreover, the Government of Shandong Province and the National Health and Family Planning Commission (formerly Ministry of Health) collaboratively launched the SMASH project in 2011, introducing interventions (i.e., promotion of LSSS and low-salt foods to consumers, and public awareness campaigns) to reduce sodium intake across the province [20]. It has shown that after five years of intervention, sales of LSSS have increased eight-fold in Shandong, which accounted for more than a quarter of sales of small-package retail salt [20].

However, the real-world use of LSSS in China and its impact on sodium and potassium intake is unclear. In this study, we used a large sample across six provinces in eastern, central, and western China to assess the awareness and use of LSSS in the natural population and to explore its association with 24-h urinary sodium and potassium excretion with the purpose of exploring whether LSSS should be promoted for salt reduction.

## 2. Materials and Methods

This was a cross-sectional study based on a pooled baseline data of three similarly designed and parallel-conducted randomized controlled trials under Action on Salt China (ASC).

### 2.1. Study Design and Participants

ASC included three similarly designed and parallel-conducted randomized controlled trials [21]: Community-based comprehensive salt reduction intervention study (CIS) [22], Home-cook salt reduction intervention study (HIS) [23], and App-based salt reduction program for primary school children and their families (AIS) [24]. Participants in these three studies were recruited from six provinces (Qinghai, Hebei, Heilongjiang, Sichuan, Jiangxi, and Hunan) in the eastern, central, and western regions of China, where no salt-related intervention studies had ever been conducted. Among these three RCTs, participants who were unable or refused to provide 24-h urine samples were excluded from this analysis. Finally, CIS involved 2693 participants in 12 rural counties, HIS involved 1576 participants in 5 rural counties and 1 urban city, and AIS involved 1184 adults in 3 urban cities. All the adult participants who completed the baseline survey were pooled together for this study.

Self-reported awareness and use of LSSS were collected using uniform questions. In CIS, only one adult was randomly selected from each family. In HIS and AIS, two adults were recruited from each family. In order to avoid reporting bias between the family members, only the family chef was selected in HIS and the adult who answered affirmatively to the LSSS awareness question was selected in AIS (i.e., “yes/no” instead of “unknown”, “yes” instead of “no”, or randomly selected if the answers were the same).

### 2.2. Survey Instrument

CIS, HIS, and AIS used the same questionnaire, which included basic information (i.e., sex, age, and education level) and 12 questions closely related to salt, covering three dimensions of knowledge (3 questions), attitude (3 questions), and behavior (6 questions). It mainly included the maximum recommended daily salt intake for adults, the relationship between sodium and salt, whether they have heard of LSSS, whether they were using LSSS, their willingness to reduce salt, and the frequency of consumption of high-salt foods (see the questionnaire in Table S1). All answers were self-reported by participants.

### 2.3. Definition of Variables

If the urine collection lasted <20 h or >28 h, the urine sample was excluded [7]. If the creatinine was <6.0 mmol for males or <4.0 mmol for females, it was also excluded [25,26]. The calculation formula of 24-h urinary sodium (potassium) excretion (mg/d) is as follows: urinary sodium (potassium) excretion (mmol/L) multiplied by 23 mg/mmol (39.1 mg/mmol). The sodium-to-potassium ratio is calculated by dividing the concentration of sodium (mmol/L) by the concentration of potassium (mmol/L) [7].

Blood pressure (BP) was measured after the participants had rested in a quiet environment for 10–15 min. Three BP readings were taken in the right arm. The average of the last two measurements were used in the analysis. Hypertension was defined as mean systolic blood pressure (SBP) ≥ 140 mm Hg or mean diastolic blood pressure (DBP) ≥ 90 mm Hg, or self-reported use of antihypertensive drugs within the last 2 weeks.

Participants were divided into “Aware” and “Unaware” groups according to the answers (yes/no) to the question “Have you ever heard of low-sodium salt?”, and the “Aware” group was further divided into “Aware and using” and “Aware but not using” group if the answer to the question “Are you using low-sodium salt?” is “Yes” or “No/Unknown”, respectively. Based on the characteristics of the data and in order to facilitate analysis and description, the participants were divided into three age groups, namely, 18–44 years old, 45–59 years old and ≥60 years old. The level of education was divided into three groups: low represented primary school education or below (0–6 years), medium represented junior high school education (7–9 years), and high represented senior high school or above (≥10 years).

### 2.4. Statistical Analysis

Mean and SD was used to describe continuous variables and frequency and percentage for categorical variables. The χ^2^ test was carried out to compare the awareness and use of LSSS among demographic groups. ANOVA was used to compare the differences in 24-h sodium and potassium excretion and sodium-to-potassium ratio among different LSSS awareness and use groups. Due to the large sample size, we used Cohen’s f to assess the effect size for ANOVA [27]. When Cohen’s f > 0.1, we considered the difference to be significant [27]. Considering that the participants might have heterogeneity in the three trials and clusters (communities), we established mixed-effect models to analyze the factors influencing the awareness and use of LSSS by defining the random effects of community levels and RCTs. Several models were established when examining the relationship between the awareness and use of LSSS and 24-h urinary sodium and potassium excretion and sodium-to-potassium ratio. All data were collated and analyzed using R 4.2.0. If *p*-value < 0.05, it could be considered that the difference is statistically significant.

## 3. Results

**Figure 1:**
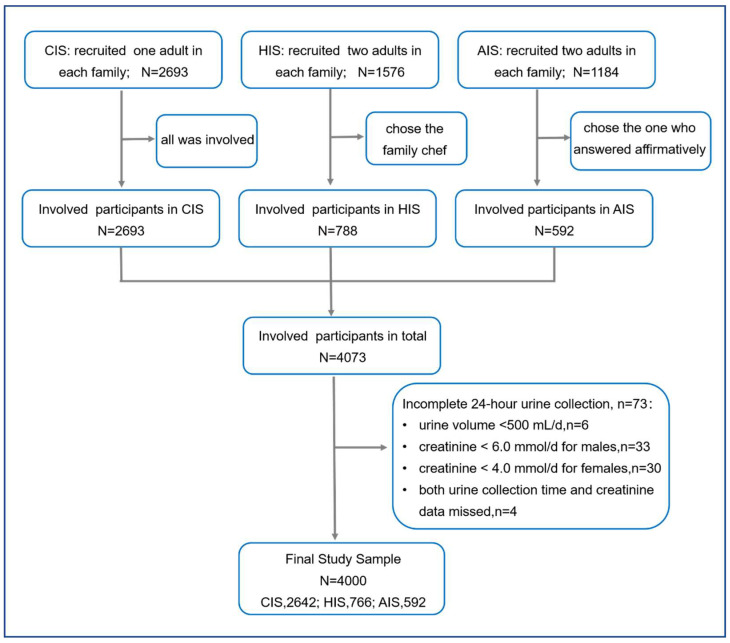
Flowchart of participants. We analyzed three independent RCTs of Action on Salt China program, including Community-based comprehensive salt reduction intervention study (CIS), Home-cook salt reduction intervention study (HIS), and App-based salt reduction program for primary school children and their families (AIS).

The three RCTs recruited 5453 participants, and 4073 participants were selected from different families. Excluding 73 participants for incomplete 24-h urine collection, we included 4000 participants in our study (Figure 1).

Of the 4000 participants, 81.9% were from rural areas, 57.3% were females, and the mean age was 49.0 (SD = 12.8) years. The average SBP and DBP were 125.8 (SD = 19.3) mm Hg and 79.2 (SD = 11.4) mm Hg and more than one-third (33.9%) had hypertension. The mean 24-h urinary sodium excretion, 24-h urinary potassium excretion and sodium-to-potassium ratio were 4333.9 (SD = 1783.7) mg/d, 1573.0 (SD = 635.8) mg/d and 5.1 (SD = 2.3), respectively (Table 1).

Among the participants, 32.0% were aware of or reported having ever heard of LSSS, 11.7% were aware of and using LSSS, while 20.3% were aware of but not using LSSS. For different demographic characteristics, participants living in cities had higher awareness and use rate of LSSS than participants living in rural areas. In terms of different age groups, the awareness and use of LSSS were the highest among participants in the 18–44 age group, which was higher than that in 45–60 and over 60 age groups. The higher the education level, the higher the awareness and use of LSSS. Participants with normal blood pressure had a higher awareness and use of LSSS than those with hypertension (all *p* < 0.001). However, there was no difference in the awareness and use of LSSS between males and females (*p* > 0.05) (Table 2).

The participants who were aware of and using or not using LSSS had better salt-related knowledge, attitude, and behavior (KAB) than those who were unaware of LSSS (Table S2).

The average 24-h urinary sodium excretion of participants who were aware of and using LSSS was 3877.1 (SD = 1416.0) mg/d, those who aware of but were not using LSSS was 4045.1 (SD = 1624.2) mg/d, and those who did not know about low sodium salt was 4498.7 (SD = 1860.3) mg/d, with a significant difference between the three groups (Cohen’s f > 0.1). However, there was no difference in the 24-h urinary potassium and sodium-to-potassium ratio among the three groups, respectively (Cohen’s f < 0.1) (Table 3).

Mixed-effect models showed that there was still a strong correlation between participants’ awareness and use of LSSS and 24-h urinary sodium after adjusting for location, sex, age, and education (*p* < 0.001). Compared with the unaware group, participants who were aware of but not using LSSS had a lower 24-h urinary sodium excretion by −356.1 (95% CI: −503.9, −205.9) mg/d. Finally, those who were aware of and using LSSS had a lower 24-h urinary sodium excretion by −490.6 (95% CI: −679.2, −293.7) mg/d (*p* < 0.001) (Table 4).

## 4. Discussion

Our study investigated the use of LSSS in real-world China and the association with 24-h urinary sodium and potassium excretion and sodium-to-potassium ratio, for the first time. Above all, nearly one-third of participants were aware of LSSS, and only 11.7% were using it at home. The use of LSSS was associated with significant reduction in 24-h urinary sodium intake by 490.6 mg/d, but not associated with significant change for the 24-h urinary potassium and sodium-to-potassium ratio.

Based on previous studies, awareness and use of LSSS varied widely in different studies. For example, the results of Chen’s survey in six regions, including Beijing, Liaoning, Hebei, Shandong, Guangzhou, and Chongqing, showed that the participants’ awareness and use of LSSS were 85.0% and 36.5% [28], while the results of YOU’s survey in the Shunyi District of Beijing showed that the participants’ awareness and use of LSSS were 41.4% and 37.4% [29], and Neal’s survey in rural areas of five provinces, including Ningxia, Shanxi, Hebei, Liaoning, and Shaanxi, showed that the participants’ awareness and use were 5.9% and 1.4%, respectively [14]. The promotion of LSSS by some cities or provinces but not by the central government might be the key reason.

Evidence has revealed that lack of knowledge, LSSS being unavailable or not easily available, with small health benefits, and higher costs were identified as barriers to the use of LSSS [29,30]. Consistent with previous studies, participants who lived in rural areas, those with low education levels, and the elderly had lower use of LSSS than others [31,32]. Moreover, the price of LSSS sold in supermarkets was about 1.5–2 times that of regular salt in China [15,33]. A previous study has shown that the use of LSSS was twice as great in villages that purchased LSSS with a subsidy as in villages without price subsidies, even given the same health education measures [34]. In China, government subsidy could be the key strategy to increase the use of LSSS.

Participants who were aware of and using LSSS reduced their 24-h urinary sodium excretion, which was in line with the findings of other studies [34]. We also found that individuals with higher awareness of LSSS tend to have more broad salt reduction knowledge and behavior, and another study also had similar finding, i.e., people having more knowledge tend to adopt healthier diet practices [35]. Additionally, Chinese cooking methods are relatively complicated. It is common that home cooks use salt-rich soy sauce and bean paste and monosodium glutamate during cooking to make the dishes colorful and tasty [36,37]. At the same time, several studies had shown that males, the young, and well-educated individuals were increasingly reliant on food cooked in restaurants [28,38,39,40]. Hence, it is difficult to measure the role of LSSS in reducing sodium intake, and the reduced 24-h urinary sodium excretion may be caused by the use of LSSS and improved salt reduction knowledge accompanied with LSSS use. A short-term cohort study could be designed to explore the association between knowledge and LSSS use, and the contribution of LSSS to the change of urinary excretion of sodium and potassium.

Contrary to other studies [34], we found no difference in 24-h urinary potassium excretion among the three groups. Based on previous studies, it is estimated that around 60% of salt comes from home cooking, and around 70% of dietary salt during home cooking comes from cooking salt [41]. Assuming that there was no other change in the diet, even if LSSS was the only salt used by a family, the increased potassium intake would be about 60% × 70% × (25%~30%) = 10%~12%, equivalent to 1680~1710 mg/d [15]. This may partially explain the lack of change in urinary potassium excretion, and implies the necessity of broad use of LSSS in most condiments and pre-packaged foods if we want to increase the population’s intake of potassium through LSSS.

In this study, the sodium–potassium ratio was lower in the ‘aware and using’ group (4.9) and ‘aware but not using’ group (4.9) when compared to the ‘unaware’ group (5.2) (*p* = 0.002 for ANOVA test), but not significant when Cohen’s f > 0.1 criteria was used (Cohen’s f = 0.055) (Table 3). To further lower the ratio, measures should be taken to further reduce population sodium intake and increase potassium intake through healthy diet and LSSS.

This study has a number of strengths. Firstly, our study pooled the data from over 4000 participants of three parallel standardized baseline surveys conducted in six provinces from eastern, central, and western China. Participants in these provinces had different eating habits, covering people with high, medium, and low salt intake. Secondly, most of the participants were recruited in rural areas, where most of the salt intake in the diet comes from salt or seasonings added during cooking, where LSSS has the greatest potential to reduce salt intake. Thirdly, the accepted gold standard of 24-h urinary sodium was used to evaluate the salt intake of the population, so the results of this study have strong accuracy. Finally, our study, for the first time, assessed the association between the use of LSSS and 24-h urinary sodium excretion in the real world.

This study has three limitations: first, the awareness and use of LSSS were self-reported by participants, which may underestimate or overestimate the actual awareness and use of LSSS. Besides, this was a cross-sectional analysis, and it was impossible to infer the causal relationship between LSSS and 24-h urinary sodium and potassium excretion, and sodium-to-potassium ratio. Nor could we measure the extent to which the reduction in 24-h urinary sodium can be attributed to LSSS.

## 5. Conclusions

This study, covering different regions of urban and rural areas in eastern, central, and western China, shows that the usage of LSSS in China is still very low, only accounting for about one-tenth of Chinese families, despite the fact that dietary sodium mainly comes from cooking salt. Together with much existing evidence and the finding that the use of LSSS was associated with reduced urinary sodium excretion in this study, the promotion of LSSS should be considered as a key salt reduction strategy in China. This is also in line with the statement in the recent WHO salt reduction report [42], that in countries where cooking salt is the main source of sodium, it is necessary to explore innovative salt reduction strategies, especially the use of LSSS.

## Figures and Tables

**Table 1:** Characteristics of participants in the study.

Characteristics	Statistics
Location, *n* (%)	
Urban ^a^	722 (18.1)
Rural	3278 (81.9)
Province, *n* (%)	
Hebei	778 (19.4)
Heilongjiang	558 (14)
Jiangxi	573 (14.3)
Hunan	756 (18.9)
Sichuan	772 (19.3)
Qinghai	563 (14.1)
Sex, *n* (%)	
Male	1710 (42.8)
Female	2290 (57.3)
Age (years), mean (SD)	49 (12.8)
Age groups (years), *n* (%)	
18–44	1588 (39.7)
45–59	1412 (35.3)
≥60	1000 (25)
Education levels, *n* (%)	
Low	1673 (41.8)
Medium	1380 (34.5)
High	947 (23.7)
Hypertension, *n* (%)	
No	2644 (66.1)
Yes	1356 (33.9)
SBP (mm Hg), mean (SD)	125.8 (19.3)
DBP (mm Hg), mean (SD)	79.2 (11.4)
Urinary collection time (h), mean (SD)	23.9 (0.4)
Urinary volume (mL/24 h), mean (SD)	1596.1 (639.4)
Urinary creatinine (mmol/24 h), mean (SD)	7490.7 (3504.6)
24-h urinary sodium (mg/d), mean (SD)	4333.9 (1783.6)
24-h urinary potassium (mg/d), mean (SD)	1573 (635.8)
Sodium-to-potassium ratio (mol/mol), mean (SD)	5.1 (2.3)

^a^: Composed of 592 urban adult participants from App-based salt reduction program for primary school children and their families (AIS) and 130 urban adults from one urban city in Home-cook salt reduction intervention study (HIS).

**Table 2:** Awareness and use of LSSS in 4000 participants with different demographic characteristics.

Characteristics	Aware of and Using LSSS	Aware of But Not Using LSSS	Unaware of LSSS	χ2	*p*-Value
Total	469 (11.7)	811 (20.3)	2720 (68.0)		
Location				509.478	<0.001
Urban	211 (29.2)	269 (37.3)	242 (33.5)		
Rural	258 (7.9)	542 (16.5)	2478 (75.6)		
Sex				5.466	0.065
Male	223 (13.0)	350 (20.5)	1137 (66.5)		
Female	246 (10.7)	461 (20.1)	1583 (69.2)		
Age groups				146.005	<0.001
18–44	258 (16.2)	418 (26.3)	912 (57.5)		
45–59	137 (9.7)	251 (17.8)	1024 (72.5)		
≥60	74 (7.4)	142 (14.2)	784 (78.4)		
Educational levels				927.616	<0.001
Low	40 (2.4)	166 (9.9)	1467 (87.7)		
Medium	138 (10.0)	289 (20.9)	953 (69.1)		
High	291 (30.7)	356 (37.6)	300 (31.7)		
Hypertension				50.247	<0.001
No	344 (13.0)	601 (22.7)	1699 (64.3)		
Yes	125 (9.2)	210 (15.5)	1021 (75.3)		

Note: LSSS stands for low-sodium salt substitutes.

**Table 3:** 24-h urinary sodium and potassium excretion, and sodium-to-potassium ratio among different LSSS awareness and use groups, mean (SD).

	24-h Urinary Sodium (mg/d)	24-h Urinary Potassium (mg/d)	Sodium to Potassium Ratio
LSSS awareness and use groups			
Aware and using	3877.1 (1416.0)	1531.9 (601.8)	4.9 (3.4)
Aware but not using	4045.1 (1624.2)	1527.6 (594.2)	4.9 (2.2)
Unaware	4498.7 (1860.3)	1593.5 (652.6)	5.2 (2.1)
Statistics for difference test among groups			
F	38.320	4.470	6.062
*p*-value	<0.001	0.012	0.002
Cohen’s f	0.138	0.047	0.055

**Table 4:** Association between LSSS and 24-h urinary sodium and potassium excretion and sodium-to-potassium ratio.

Variables	24-h Urinary Sodium		24-h Urinary Potassium		Sodium to Potassium Ratio	
	β (95% CI)	*p-Value*	β (95% CI)	*p-Value*	β (95% CI)	*p-Value*
LSSS group (Ref = ‘Unaware of’)						
Aware but not using	−356.1 (−503.9, −205.9)	<0.001	−61.2 (−115.4, −7.0)	0.027	−0.2 (−0.4, −0.0)	0.029
Aware and using	−490.6 (−679.2, −293.7)	<0.001	−52.8 (−122.6, 16.9)	0.138	−0.2 (−0.4, 0.1)	0.253
Location (Ref = ‘Urban’)						
Rural	754.9 (157.4, 1055.5)	<0.001	−12.2 (−68.8, 55.8)	0.674	0.3 (0.1, 0.6)	0.003
Sex (Ref = ‘Male’)						
Female	−505.9 (−623.8, −387.6)	<0.001	29.2 (−11.8, 70.1)	0.163	−0.7 (−0.8, −0.5)	<0.001
Age groups (Ref = ‘18–44’)						
45–59	−120.9 (−256.1, 8.6)	0.073	88.4 (41.1, 135.6)	<0.001	−0.5 (−0.7, −0.3)	<0.001
≥60	−492.7 (−647.4, −342.1)	<0.001	−13.2 (−67.6, 41.3)	0.636	−0.6 (−0.8, −0.4)	<0.001
Education levels (Ref = ‘Low’)						
Medium	−132.9 (−270.0, 0.0)	0.054	19.8 (−29.2, 68.9)	0.429	−0.2 (0.4, −0.0)	0.050
High	−305.6 (−480.4, −132.9)	<0.001	−9.1 (−72.4, 54.2)	0.779	−0.51 (−0.5, −0.1)	0.007

## Data Availability

Relevant anonymized individual level data will be made available one year after publication of the primary manuscript on request from the corresponding author. Request for data sharing will be handled in line with the relevant regulations for data access and sharing in China and will need the approval of the trial steering committee and Institutional Review Board of the Chinese Center for Disease Control and Prevention.

## Supplementary Materials

The following supporting information can be downloaded at: https://www.mdpi.com/article/10.3390/nu15133000/s1, Table S1: The questionnaire used by CIS, HIS and AIS, the three randomized controlled trials underpinning the study; Table S2: Knowledge, attitude and behavior (KAB) towards salt reduction among people with different LSSS awareness and using status.
