# Supplementary material for: Awareness and Use of Low-Sodium Salt Substitutes and Its Impact on 24-h Urinary Sodium and Potassium Excretion in China—A Cross-Sectional Study
Source: Nutrients. 2023 Jun 30;15(13):3000. doi: 10.3390/nu15133000 (PMC10346169; doi:10.3390/nu15133000)
Supplement: Supplementary file 1 [file nutrients-15-03000-s001.zip › nutrients-2470108-supplementary.pdf]

Supplementary Materials

Table S1: The questionnaire used by CIS, HIS and AIS, the three randomized controlled trails underpinning the study

Questions
Knowledge (3 questions)
Do you know what is the recommended maximum level of salt per day?
Less than 2 grams
Less than 5 grams
Less than 8 grams
Less than 12 grams
More than 12 grams
Don’t know
Have you heard of low sodium salt?
Yes
No
Which item on this food label represents the salt content?
Energy
Protein
Fat
Carbohydrates
Sodium
Don’t know
Attitudes (3 questions)
Do you agree high salt intake would cause hypertension?
Agree
Disagree
Don’t know
Do you agree low salt intake would make people limb weakened?
Agree
Disagree
Don’t know
Would you like to choose low-salt diet?
Yes
No
Don’t know
Behaviors (6 questions)
What is your usual taste for food?
More Salty
Moderate
Less Salty
Do you use low-sodium salt at home?
Yes

No
Don’t know
How often have you consumed pickled foods in the past month?
Almost every day
3-5 days per week
1-2 days per week
Once per week or less
How often have you consumed salty snacks in the past month?
Almost every day
3-5 days per week
1-2 days per week
Once per week or less
How often did you eat out or order delivery in the past month?
Almost every day
3-5 days per week
1-2 days per week
Once per week or less
Never
Have you requested for less-salted meals when eating out in the past month?
Always
Sometimes
Occasionally
Never

Table S2: Knowledge, attitude and behavior (KAB) towards salt reduction among people with different LSSS awareness and using status

Questions	Aware of and using LSSS	Aware of but not using LSSS	Unaware of LSSS	$\chi 2$	p-value
Know the recommended maximum level of salt per day	217 (46.3) [a]	234 (36.6) [b]	427 (14.8) [c]	328.660	<0.001
Know the nutrition label of salt	369 (78.7) [a]	404 (63.1) [b]	896 (31.0) [c]	520.873	<0.001
Know that eat too much salt will lead to hypertension	413 (88.1) [a]	529 (82.7) [b]	2101 (72.7) [c]	70.616	<0.001
Agree low salt intake wouldn't make people limb weakened	185 (39.4) [a]	244 (38.1) [a]	698 (24.1) [b]	83.964	<0.001
Willing to choose low-salt diet	423 (90.2) [a]	552 (86.3) [a]	2311 (79.9) [b]	37.670	<0.001
What is your usual taste for salt?					
Prefer salty food	125 (26.7) [a]	159 (24.8) [a]	872 (30.2) [a]	23.692	<0.001
No obvious preference	225 (48.0) [a]	331 (51.7) [a]	1211 (41.9) [b]		

Prefer less salty food	119 (25.4) [a]	150 (23.4) [a]	808 (27.9) [a]		
How often have you consumed pickled foods in the past month?					
Almost every day	18 (3.8) [a]	57 (8.9) [b]	406 (14.0) [c]	59.970	<0.001
3-5 days per week	26 (5.5) [a]	44 (6.9) [a]	248 (8.6) [a]		
1-2 days per week	96 (20.5) [a]	125 (19.5) [a]	570 (19.7) [a]		
Once per week or less	329 (70.1) [a]	414 (64.7) [a]	1667 (57.7) [b]		
How often have you consumed salty snacks in the past month?					
Almost every day	2 (0.4) [a]	7 (1.1) [a]	16 (0.6) [a]	24.982	<0.001
3-5 days per week	9 (1.9) [a]	13 (2.0) [a]	29 (1.0) [a]		
1-2 days per week	42 (9.0) [a]	55 (8.6) [a]	156 (5.4) [b]		
Once per week or less	416 (88.7) [a]	565 (88.3) [a]	2690 (93.0) [b]		
How often did you eat out or order delivery in the past month?					
Almost every day	16 (3.4) [a]	30 (4.7) [a]	94 (3.3) [a]	73.742	<0.001
3-5 days per week	51 (10.9) [a]	49 (7.7) [a,b]	153 (5.3) [b]		
1-2 days per week	97 (20.7) [a]	106 (16.6) [a]	328 (11.3) [b]		
Once per week or less	251 (53.5) [a]	363 (56.7) [a]	1872 (64.8) [b]		
Never	54 (11.5) [a]	92 (14.4) [a]	444 (15.4) [a]		
Have you requested for less-salted meals when eating out in the past month?					
Always	107 (22.8) [a]	136 (21.3) [a]	579 (20.0) [a]	26.725	<0.001
Sometimes	33 (7.0) [a]	38 (5.9) [a]	110 (3.8) [b]		
Occasionally	30 (6.4) [a]	37 (5.8) [a]	117 (4.0) [a]		
Never	299 (63.8) [a]	429 (67.0) [a]	2085 (72.1) [b]		

LSSS: low-sodium salt substitutes. Footnotes of a, b and c indicate the significance of pairwise comparison. If the footnotes are the same for any two groups, the difference between the two groups is not statistically significant; if the footnotes are different, the difference between groups is statistically significant.
